# A dual-pathway Wnt-IL-13 fusion protein enhances human intestinal regeneration through tuft cell activation

**DOI:** 10.1016/j.jbc.2026.113187

**Published:** 2026-05-24

**Authors:** Li Yang, Lulu Huang, Fenna L.H. van Rijt, Baojie Zhang, Amir Giladi, Jochem H. Bernink, Hans Clevers, Claudia Y. Janda

**Affiliations:** 1 The Princess Máxima Center for Pediatric Oncology, Utrecht, the Netherlands; 2 Hubrecht Institute, Royal Netherlands Academy of Arts and Sciences (KNAW) and University Medical Center Utrecht, Utrecht, the Netherlands; 3 Oncode Institute, Utrecht, the Netherlands; 4 Amsterdam University Medical Center, University of Amsterdam, Department of Experimental Immunology, Amsterdam Institute for Immunology and Infectious Diseases, Amsterdam, the Netherlands

**Keywords:** intestinal regeneration, tuft cells, human intestinal organoids, Wnt signaling, IL-13 signaling, Wnt surrogate

## Abstract

Compromised intestinal regenerative responses drive severe inflammatory conditions, such as inflammatory bowel disease and graft-*versus*-host disease, affecting millions of people worldwide each year. Despite extensive research, effective therapies remain limited, and no curative treatments are currently available. We recently discovered that human intestinal tuft cells promote tissue repair following injury through Wnt and IL-4/IL-13 signaling pathways. Building on this discovery, here, we report the engineering and functional validation of a synthetic Wnt-IL-13 fusion protein that simultaneously activates both the Wnt and IL-4/IL-13 signaling pathways to enhance human intestinal tuft cell activity. Employing human organoid technology, we demonstrate that this therapeutic approach promotes mucosal healing.

Continuous intestinal epithelial renewal is essential to maintain or restore epithelial integrity in case of injury, preventing bacterial dissemination and uncontrolled inflammation. This process is orchestrated primarily by intestinal stem cells (ISCs) and transit-amplifying cells , which continuously replenish the epithelium (1, 2). A coordinated network of growth factors and morphogens acts at the bottom of intestinal crypts on ISCs to preserve stemness and drive continuous epithelial proliferation (3, 4, 5). Disruption or loss of ISCs delays epithelial renewal and compromises barrier integrity. As a consequence, bacterial translocation into otherwise sterile mucosal tissues can occur, triggering severe inflammatory responses. Such compromised mucosal integrity drives multiple clinical conditions, such as intestinal mucositis in leukemic patients who undergo preconditioning regimen prior to receiving a hematopoietic stem cell graft (6), or severe intestinal ulcers in patients who suffer from inflammatory bowel disease (7). While disease severity in such patients is assessed by various endoscope-based scoring systems that center on mucosal healing, current treatment options for intestinal inflammation are primarily aimed at suppressing inflammation rather than boosting the epithelial restorative response itself.

Recent insights into human mucosal regenerative biology have identified tuft cells as a rare epithelial cell type with epithelial reparative properties (8). Mechanistically, tuft cell development and proliferation critically depend on the Wnt and interleukin-4 (IL-4)/interleukin-13 (IL-13) signaling pathways. Upon activation, tuft cells can act as a local cellular source of growth factors such as epiregulin and heparin-binding EGF-like growth factor, and can transdifferentiate into ISCs, thereby contributing to tissue regeneration. Given the roles of Wnt and IL-4/IL-13 signaling in promoting intestinal regeneration, simultaneous therapeutic activation represents a promising approach, though it faces several obstacles.

Wnt ligands activate signaling by binding to Frizzled (FZD) and low-density lipoprotein receptor-related protein(LRP)-5/6 receptors. The 10 FZDs (FZD1-10) exhibit distinct yet overlapping expression patterns across tissue cell types, though their individual contributions to regenerative responses remain incompletely understood. A major limitation for therapeutic development is that Wnt proteins require lipid modification for receptor binding, rendering them highly hydrophobic and unsuitable for drug development (9, 10). Early work by Cong and colleagues demonstrated that ligand-induced crosslinking of FZD and LRP5/6 can activate Wnt/β-catenin signaling (11). Building on this concept, subsequent work showed that enforced proximity of FZD and LRP5/6 receptors, using cross-linked antagonists that block Wnt binding to both receptors, provided direct evidence that receptor crosslinking alone is sufficient to drive pathway activation, forming the basis for the development of antibody-based Wnt surrogates (12, 13, 14). These engineered bispecific ligands promote FZD-LRP5/6 heterodimerization, effectively mimicking natural Wnt ligands while circumventing the challenges of working with native Wnt proteins. As a result, various Wnt surrogates are being developed as potential regenerative therapeutics (15, 16, 17, 18, 19).

Similarly, therapeutic activation of the IL-4/IL-13 axis presents both opportunities and challenges. These cytokines, which are crucial for regulating immune responses and tissue repair, signal through type I and type II receptor complexes involving IL-4 receptor alpha (IL-4Rα) paired with either the common gamma chain, or IL-13 receptor alpha 1 (IL-13Rα1), respectively (20). While IL-4 can engage both type I and type II receptor complexes, IL-13 is restricted to signaling through the type II receptor complex. The widespread expression of IL-4/IL-13 receptors across diverse cell types underlies their central role in type 2 immune responses, including Th2 cell differentiation, M2 macrophage polarization, and eosinophil recruitment (21). However, this broad receptor distribution complicates therapeutic applications, as systemic administration risks off-target effects including uncontrolled inflammation, excessive mucus production, and tissue fibrosis. Consequently, strategies that spatially restrict IL-4/IL-13 signaling to the epithelium while minimizing systemic exposure could enhance safety profiles for regenerative applications.

Here, we leverage human intestinal organoid models to explore a novel class of therapeutic strategies aimed at promoting mucosal healing by targeting tuft cells. Specifically, we engineered a fusion protein that simultaneously activates Wnt and IL-13 signaling to boost the regenerative properties of human intestinal tuft cells. We demonstrate a proof-of-concept approach for the development of alternative therapeutics that primarily act to enhance epithelial restoration rather than solely suppress inflammation.

## Results

### Wnt and IL-4/IL-13 receptor expression profiles in tuft cells inform the engineering strategy of Wnt-IL-13 fusion proteins

In a previous study (8), tuft cells were shown to express high levels of IL-13Rα1 and, to a lesser extent, IL-4Rα in human intestinal tissues, making IL-13 the cytokine of choice for our study. Furthermore, its more restricted receptor specificity and reduced pleiotropy confer a narrower and more defined target cell range than that of IL-4. To design an appropriate engineering strategy for a Wnt–IL-13 fusion protein, we first characterized the expression profiles of Wnt receptors across human intestinal epithelial lineages, with particular focus on tuft cells. Specifically, we profiled the expression of the 10 FZDs and LRP5/6 coreceptors using a previously generated single-cell RNA sequencing (scRNA-seq) dataset from ileum organoid-derived epithelial cells cultured in tuft cell differentiation medium, with and without exposure to IL-4/IL-13 (8). This dataset contained four distinct tuft cell subpopulations, in addition to stem cells and two goblet cell types.

**Figure 1:**
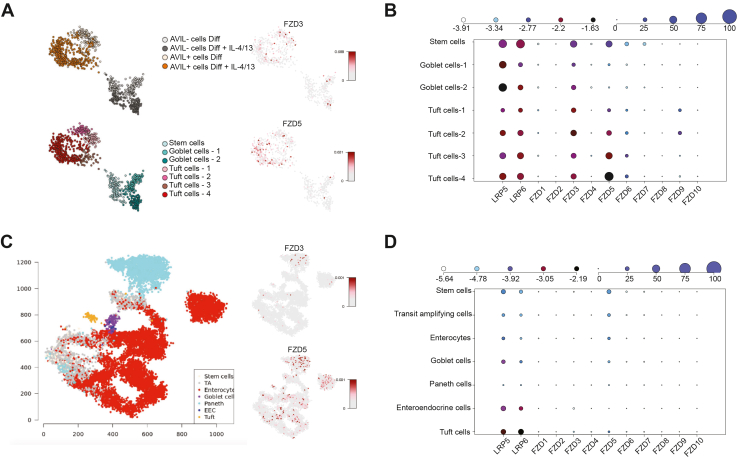
**Wnt and IL-4/IL-13 receptor expression profiles in human intestinal epithelial cells.***A*, Metacell 2D representation of scRNA-seq data of adult human ileum AVIL-Clover reporter organoids. Organoids were cultured in human intestinal expansion medium for 4 days, then differentiated for another 4 days in tuft cell differentiation medium with or without IL-4/IL-13 treatment, and sorted based on AVIL expression before sequencing (8). n = 953 single cells, colored by medium conditions (*top left*), cell types (*bottom left*), as well as the log-normalized expression of FZD3 and FZD5 (*right*). Diff: human intestinal tuft cell differentiation medium. *B*, Expression of the Wnt receptors FZD and LRP5/6 across the scRNA-seq dataset in (*A*). Dot color relates to size-normalized mean expression values and dot size to fraction of expressing cells. n = 953 single cells. *C*, Metacell 2D representation of scRNA-seq data of epithelial cells derived from primary human adult small intestine (22). n = 15,184 single epithelial cells colored by their cell subtypes (*left*), as well as log-normalized expression of FZD5 and FZD3 (*right*). *D*, Expression of the Wnt receptors FZD and LRP5/6 across the scRNA-seq dataset in (*C*). Dot color relates to size-normalized mean expression values and dot size to fraction of expressing cells. FZD, Frizzled; LRP, lipoprotein receptor-related protein; IL, interleukin; IL-13Rα1, IL-13 receptor alpha 1; IL-4Rα, IL-4 receptor alpha; scRNA-seq, single-cell RNA sequencing.

Across the four tuft cell subsets, FZD5 and FZD3 were strongly expressed, with moderate expression of FZD6 (Figs. 1, *A*, *B* and S1). FZD9 expression was more restricted, predominating in the tuft-1 and tuft-2 subsets. Notably, FZD5, FZD3, and FZD6 were also detected in stem and goblet cells, suggesting broader functions for these receptors beyond the tuft cell lineage. Both LRP5 and LRP6 were consistently expressed across all tuft subsets. To validate these observations, we analyzed an independent scRNA-seq dataset of primary human adult small intestine (22) (Figs. 1, *C*, *D* and S2). The expression pattern in primary tissue was more heterogeneous; however, LRP5 and LRP6 remained strongly expressed in tuft cells. FZD5 again emerged as the predominant FZD receptor, albeit at lower levels than in the tuft cells in organoids as well as all other cells within the intestinal epithelial lineage, spanning stem cells, transit-amplifying cells, enterocytes, goblet cells, and enteroendocrine cells. Taken together, these data thus provide a rationale for developing Wnt-IL-13 fusion proteins that engage FZD5 to drive robust activity or FZD9 to achieve greater selectivity for tuft cells.

### Engineering of trispecific Wnt-IL-13 fusion proteins for the simultaneous activation of Wnt and IL-13 signaling

**Figure 2:**
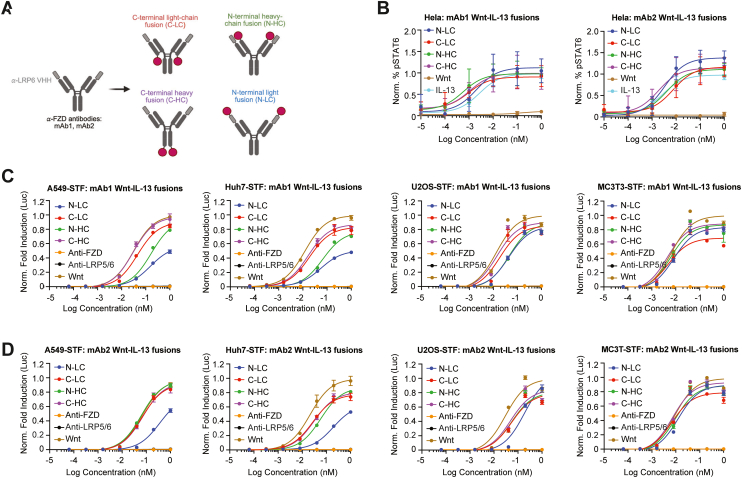
**Functional characterization of trispecific Wnt–IL-13 fusion proteins.***A*, schematic representation of the trispecific Wnt–IL-13 fusion protein formats. The anti-FZD IgG antibodies are shown in *black*, the anti-LRP5/6 VHH domain in *gray*, and IL-13 as a *red circle*. Created with BioRender.com. *B,* STAT6 phosphorylation induced by IL-13 and the Wnt–IL-13 fusion proteins in HeLa cells, measured by FACS. The percentage of pSTAT6-positive cells in stimulated samples minus unstimulated samples was normalized relative to the pSTAT6 level observed with recombinant IL-13. Data represent mean ± SD of three biological replicates, each containing the mean of at least two technical replicates. *C*–*D*, activation of the β-catenin–dependent STF reporter by Wnt surrogates (mAb1 and mAb2) and Wnt–IL-13 fusion proteins in A549 (expresses predominantly FZD2 > FZD6 > FZD7 > FZD3), Huh7 (FZD5 > FZD4 > FZD7 > FZD6 > FZD1), U2OS (FZD6 > FZD3 > FZD1 > FZD7), and MC3T3 cells (FZD1 > FZD7). Cells were stimulated with Wnt surrogates (mAb1- and mAb2-based) or Wnt-IL-13 fusion proteins, and luciferase activity is shown as fold induction relative to unstimulated samples and normalized to the corresponding Wnt surrogate. Data represent mean ± SD of three biological replicates, each containing the mean of three technical replicates. FZD, Frizzled; pSTAT6, STAT6 phosphorylation; STF, SuperTOPFlash.

To test whether tuft cells can be expanded through simultaneous activation of Wnt and IL-13 signaling, we generated a panel of Wnt–IL-13 fusion proteins aimed at robustly and simultaneously activating Wnt and IL-13 signaling within the same cell. These constructs combined previously validated Wnt surrogate formats, comprising anti-FZD antibodies and a single-domain variable domain (variable domain of the heavy chain of heavy-chain-only antibody [VHH]) that binds LRP5/6 fused to the N terminus of the light chains (LCs), with WT human IL-13 connected *via* a flexible peptide linker (Fig. 2*A*) (14). We took advantage of publicly available antibodies and antibody fragments. Two anti-FZD antibodies were selected based on their binding to FZD5: mAb1, which binds FZD1/2/5/7/8 (23), and mAb2, which binds FZD1/2/4/5/7/8 (24); and an LRP6-binding VHH (25).

Because the addition of IL-13 could sterically interfere with receptor binding by either the Wnt surrogate (to FZD and LRP5/6) or IL-13 (to IL-13Rα1 and IL-4Rα), IL-13 was fused to four different positions, either the N or C terminus of the heavy chain (HC) or LC of the anti-FZD antibody, to identify a geometry that optimizes dual signaling. All constructs, including control Wnt surrogates and antibodies, were transiently expressed in Expi293F cells and purified by protein A affinity chromatography followed by size-exclusion chromatography (SEC) (Figs. S3 and S4).

We first examined whether the fusion proteins retained IL-13 signaling activity by measuring STAT6 phosphorylation (pSTAT6) in HeLa cells, which express IL-13 receptors but display low basal STAT6 activation. All Wnt–IL-13 formats induced pSTAT6 with similar potency to recombinant IL-13, independent of fusion orientation, indicating that IL-13 activity was preserved (Fig. 2*B*).

Next, we assessed Wnt signaling activity using the SuperTOPFlash (STF) reporter assay, a standard readout of Wnt/β-catenin signaling, in several cell lines (A549, Huh7, U2OS, MC3T3) with distinct FZD expression patterns (Fig. 2, *C* and *D*). In the mAb1 context, C-terminal IL-13 fusions to either the LC (C-LC) or heavy chain (C-HC) showed comparable activity to the parental Wnt surrogate, whereas N-terminal fusions (N-LC, N-HC) displayed reduced signaling in A549 and Huh7 cells but comparable activity to the positive control in U2OS or MC3T3 cells. In the mAb2 context, only the Wnt-IL-13 fusion in which IL-13 was linked to the N terminus of the LC consistently showed lower activity than that of the Wnt surrogate itself, across cell types. All others (C-LC, C-HC, and N-HC) showed comparable activity to the Wnt surrogate.

Together, these data indicate that C-terminal fusions of IL-13 generally preserve Wnt and IL-13 signaling activity more effectively than N-terminal fusions, likely by minimizing steric hindrance between the IL-13 ligand and the Wnt surrogate domains. This panel of engineered Wnt–IL-13 fusion proteins provides a versatile platform to study the coordinated activation of Wnt and IL-13 signaling pathways.

### Wnt-IL-13 fusion protein enhances human intestinal tuft cell regeneration

**Figure 3:**
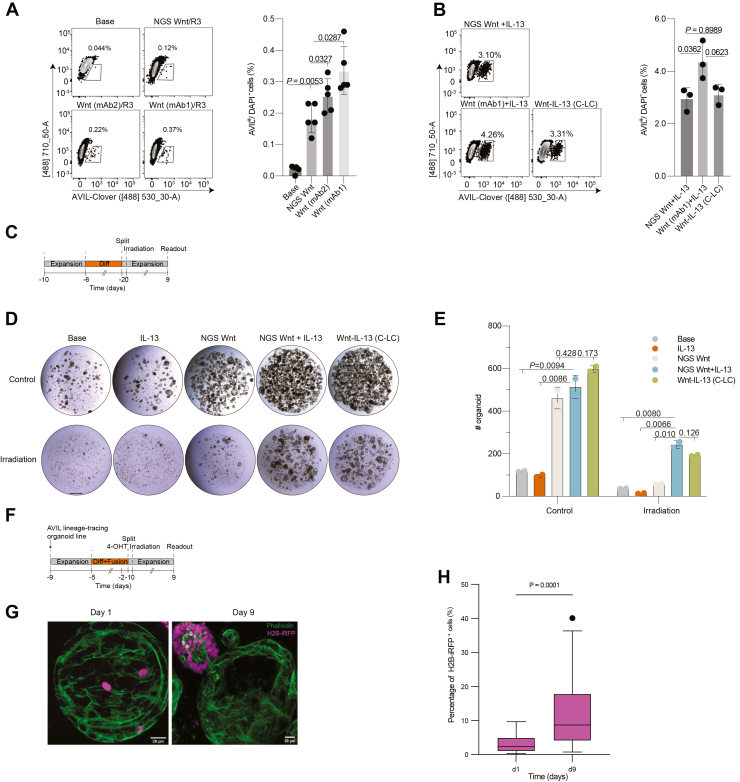
**Wnt-IL-13 fusion protein in human intestinalorganoids.***A*-*B*, representative flow cytometric analysis (*left*) and quantification (*right*) of tuft (AVIL^+^) cell percentage of the single live (DAPI^−^) cells in human ileum AVIL-Clover reporter organoids. Organoids were cultured in standard human intestinal expansion medium for 4 days and then differentiated for another 5 to 10 days in tuft cell differentiation medium with indicated recombinant proteins. Each dot is a well. *A*, *n* = 5 wells per condition (pooled from three independent experiments). *B*, *n* = 3 wells per condition, results are representative of three independent experiments. *C*–*E*, schematic (*C*), representative images (*D*), quantification of organoid numbers (*E*) from adult human ileum organoids following irradiation. After growing in human intestinal expansion medium for 4 days, human ileum organoids were differentiated for another 4 days in tuft cell differentiation medium with the indicated proteins. Then, organoids were split and irradiated at 6 Gy. The organoids were allowed to recover for 9 days in expansion medium after irradiation. Experiments were performed on two clones (Fig. S3*B*). *E*, each dot is 1 BME drop. *n* = 2 drops per condition. *F*–*H*, schematic (*F*), representative images (*G*), and quantification (*H*) of tuft cell (AVIL) lineage-tracing organoids after irradiation. AVIL lineage-tracing organoids were differentiated in tuft cell medium with fusion protein for 4 days, exposed to 1 μM 4-OHT for 20 h, split and irradiated at 9 Gy. The organoids were allowed to recover for 9 days in expansion medium after irradiation. *H*, results are pooled from three independent experiments, *n* = 26 (day 1) or 32 (day 9) organoids. *A*, *B*, and *E*, data are presented as mean ± SD. *H*, box plots show data from the 25th to 75th percentiles with whiskers extending to the minimum and maximum within 1.5 × interquartile range, with dots marking outliers. *A*, *B*, and *E*, *p* values are derived from one-way ANOVA with Dunnett’s test for multiple comparisons against the Wnt(UPE)/R3 group (*A*) or one-way ANOVA with Tukey's multiple comparisons test (*B*). *E*, *p* values are derived from false discovery rate (FDR)-adjusted unpaired two-tailed Student’s *t* test against the NGS Wnt + IL-13 group. *H*, *p* values are derived from two-tailed Student’s *t* test. Scale bars represent 1 mm (*D*) and 20 μm (*G*). DAPI, 4′,6-diamidino-2-phenylindole; IL-13, interleukin-13; R3, R-spondin3.

We previously took advantage of human organoid technology to systematically dissect niche factors and requirements for tuft cell development and proliferation. Building on these insights, we employed our engineered human intestinal tuft cell reporter organoids (8) to assess the activity of two Wnt-IL-13 fusion proteins, with IL-13 fused to the C terminus of the LC of mAb1 and mAb2 (Fig. S5*A*). To identify the most promising Wnt-IL-13 fusion protein for activating intestinal tuft cells, we took a stepwise approach. We first compared the activities of the Wnt surrogates based on mAb1 or mAb2 with the commercial Wnt Surrogate-Fc, referred to as next generation surrogate [NGS] Wnt. Relative to base medium lacking Wnt, tuft cell frequency increased approximately 7-fold in the presence of NGS Wnt. The mAb2 and mAb1-based Wnt surrogates showed a 5- and 8-fold increase, respectively, over baseline (Fig. 3*A*). The difference in activity may be related to intrinsic differences of mAb1 *versus* mAb2, such as affinities, rather than related to the FZD binding specificities of the mAb1/2 themselves. Based on its superior activity, subsequent experiments focused on the mAb1-based Wnt-IL-13 fusion protein with IL-13 fused to the C terminus of the LC.

Consistent with prior findings that the addition of recombinant IL-13 promotes tuft cell proliferation (8), reporter organoids cultured with NGS Wnt plus IL-13 triggered a 20-fold increase in tuft cell frequency as compared to NGS Wnt alone. This effect was even more pronounced with the mAb1-based Wnt surrogate plus IL-13 (Fig. 3*B*). Notably, tuft cell reporter organoids cultured with the Wnt-IL-13 (C-LC) fusion protein expanded comparably to those treated with IL-13 plus mAb1-based Wnt surrogate, demonstrating the potency of a single synthetic protein to induce tuft cell expansion in human intestinal organoids (Fig. 3*B*).

To test whether Wnt-IL-13 (C-LC) promotes tuft cell–mediated regeneration in a setting of epithelial injury, we irradiated differentiated human intestinal tuft cell reporter organoids and monitored recovery over a 9-day period (Fig. 3*C*). Organoids treated with NGS Wnt or IL-13 alone failed to recover, whereas organoids exposed to either combined compounds or Wnt-IL-13 (C-LC) successfully regained growth capacity (Figs. 3, *D*, *E* and S5*B*). To corroborate these findings, we performed experiments with tuft cell lineage-tracing organoids, subjected to irradiation and recovery (Fig. 3*F*). Wnt-IL-13 (C-LC) treatment restored organoid growth and resulted in tuft cell–derived organoid segments (infrared red fluorescent protein^+^ cells), along with an overall increase in infrared red fluorescent protein-labeled cell frequency (Fig. 3, *G* and *H*).

Together, these findings demonstrate that the engineered Wnt-IL-13 fusion protein potently activates tuft cells and induces a regenerative response in compromised human intestinal epithelium *in vitro*.

## Discussion

In this study, we leveraged human intestinal organoid technology to demonstrate that human intestinal tuft cells are therapeutically targetable epithelial cells with potent regenerative capacity. We show that a synthetic fusion protein with drug-like properties that coactivates Wnt and IL-13 signaling enhances the regenerative response of the human intestine after radiation injury by engaging tuft cell–mediated repair. These findings build on our previous work demonstrating that loss of canonical ISCs can be compensated by activation of tuft cells, a rare epithelial population positioned at the crypt–villus junction, and that their reparative function can be amplified through concurrent activation of Wnt and IL-13 signaling. A key finding of this study is the identification of a fusion geometry, linking IL-13 to the C terminus of either the HC or LC of the antibody-based Wnt surrogate, that preserves the function of both signaling arms. Beyond efficacy, combining dual pathway activation within a single molecule simplifies pharmacokinetics and manufacturing and may allow for more spatially restricted IL-13 signaling compared to free IL-13. Together, these results provide the first translational demonstration that targeted activation of tuft cell–driven repair with a single biologic can be harnessed to restore mucosal integrity, for instance in inflammatory and cytotoxic injury settings.

Current therapies for the management of intestinal inflammatory conditions that emerge as a consequence of severe mucosal barrier disruption primarily aim to suppress the inflammatory cascade, while a focus on adequately promoting mucosal healing is lacking. Our work demonstrates that selectively boosting tuft cell activity may provide a complementary and potentially synergistic strategy that directly enhances epithelial repair. The Wnt-IL-13 fusion protein described here may therefore serve as a prototype for therapies designed to restore, rather than merely preserve, intestinal function, by simultaneously activating the Wnt and IL-13 signaling pathways, without the need for providing the individual agonists exogenously. Nevertheless, our approach has several limitations and will benefit from further refinement. Most notably, while the engineering strategy of Wnt-IL-13 fusion proteins could in principle enable selective targeting and localized activity, the current construct is not designed for such specificity. The Wnt surrogate used here engages broadly expressed FZD receptors, resulting in a cross-reactive profile rather than cell type–restricted targeting. As a consequence, selective targeting and potential off-target or systemic effects have not yet been addressed and will require further engineering and evaluation in future studies. These considerations align with broader gaps in the translational pipeline for IL-13 and Wnt-based therapies and are mostly related to their inherent biochemical properties and pleiotropic activities.

The clinical translation of IL-13 and IL-4 agonists as therapeutic agents remains largely unexplored. Most insights stem from preclinical models investigating signaling through the type II receptor complex (IL-4Rα/IL-13Rα1), which is predominantly expressed on nonhematopoietic cells such as epithelial, stromal, and tumor cells (21). In these models, IL-4 and IL-13 have been shown to promote wound healing and tissue repair, highlighting opportunities in regenerative medicine. However, their dual nature poses major clinical challenges, as the same pathways that mediate tissue regeneration also drive pathological fibrosis and allergic inflammation. Engineered cytokine variants, including IL-4 superkines and IL-13 analogs with altered receptor-binding specificity, offer a way to harness their beneficial effects while limiting adverse outcomes (26, 27). In particular, IL-13 variants with increased affinity for the IL-13Rα1 receptor chain present a promising avenue for further exploration in the context of the Wnt-IL-13 fusions. Moreover, the clinical development of cytokine therapeutics faces additional hurdles, most notably suboptimal pharmacokinetic and stability profiles, challenges that are actively being addressed through the design of the Wnt-IL-13 fusion proteins.

Furthermore, the natural Wnt ligands represent powerful modulators of tissue repair and regeneration, and the development of Wnt surrogate technology has addressed the limitation of the unfavorable developability of Wnt ligands as therapeutic agents by demonstrating that forced heterodimerization of the Wnt receptors FZDs and LRP5/6 is sufficient to activate β-catenin–dependent Wnt signaling, thereby bypassing the need for the natural lipidated ligand (12, 13). Several Wnt surrogates have since been developed for systemic delivery, showing efficacy in promoting tissue regeneration and repair in preclinical models of bone (osteoporosis, nonunion fracture), intestine, and liver injury (14). Despite this progress, clinical translation remains challenging. The only reported clinical trial involving systemic administration of an antibody-based Wnt surrogate for inflammatory bowel disease was discontinued after phase I due to elevated liver enzyme levels, suggestive of potential hepatotoxicity. Hence, future work on optimizing the activity of the Wnt-IL-13 fusion protein could explore strategies to confine its activity specifically to the intestine. One avenue could involve engineering tetra-specific variants that additionally target tuft cells by incorporating a binding domain for a tuft cell–specific receptor. Alternatively, scRNA-seq data from human intestinal tissue indicate that FZD9 is most differentially expressed in tuft cells. FZD9 displays a more restricted expression pattern compared to the broadly expressed receptors FZD1/2/5/7/8. Notably, while most current anti-FZD antibodies target FZD1/2/5/7/8, redirecting specificity toward FZD9 could further enhance selectivity for tuft cells and minimize off-target effects.

A limitation of this study is that functional validation was performed exclusively in human intestinal organoid models. While these provide a human-specific system that captures intestinal epithelial biology and are well suited for establishing proof-of-concept, they do not recapitulate the full complexity of the intestinal niche and lack key components, including endothelial, stromal, and immune cells that contribute to regeneration *in vivo*. Importantly, direct *in vivo* validation is not straightforward, as human IL-13 does not sufficiently engage murine IL-13 receptors, precluding the use of standard mouse models. In addition, differences between mouse and human tuft cell populations may further limit the relevance of murine systems for this pathway (8, 28). Future studies will therefore require alternative approaches, including advanced coculture or organ-on-chip systems, as well as humanized mouse models expressing human IL-13 receptors or, where appropriate, nonhuman primate models, to further assess translational relevance.

## Experimental procedures

### Expression and purification of Wnt-IL-13 variants and control antibodies

The coding sequences of the Wnt–IL-13 variants, anti-FZD antibodies, and anti-VHH–Fc variants (for sequences, see Appendix A) were cloned into the pcDNA3.1 vector containing an N-terminal signal peptide. Proteins were expressed by transient transfection of Expi293F cells (Gibco; Thermo Fisher Scientific) using the ExpiFectamine 293 Transfection Kit (Gibco; Thermo Fisher Scientific), following the manufacturer’s instructions. After 4 days of expression, the conditioned media were clarified by centrifugation, and the secreted proteins were captured using CaptivA protein A affinity resin (Repligen). Bound proteins were eluted with IgG elution buffer (Thermo Fisher Scientific) containing 300 mM NaCl and immediately neutralized with 100 mM Tris (pH 8.0) to reach a final pH of 7.0. Subsequently, proteins were further purified by SEC on a Superdex 200 Increase 10/300 GL column (Cytiva) using an ÄKTA Pure chromatography system (Cytiva). The running buffer consisted of 1 × HBS (20 mM Hepes, pH 7.3; 300 mM NaCl). Fractions obtained after SEC were analyzed by SDS-PAGE (4–20% gradient gel, Bio-Rad) and stained with InstantBlue Coomassie Protein Stain (Abcam). Purified proteins were concentrated using Amicon Ultra centrifugal filters (Millipore; molecular weight cut-off 30 kDa) and sterilized by filtration through 0.22 μm centrifugal filters (Merck). Protein concentrations were determined by NanoDrop A280 measurements using the respective molecular weights and extinction coefficients.

### Cell lines for measuring signaling activities

A549, Huh7, MC3T3, and U2OS cell lines, stably transfected with the Wnt-responsive STF reporter plasmid, were a kind gift from the laboratory of Prof. Dr. K. Christopher Garcia (Stanford University). HeLa cells were purchased from American tyoe culture collection. A549 cells were cultured in RPMI-1640 medium (Gibco); Huh7 and HeLa cells in high-glucose Dulbecco's modified Eagle's medium (DMEM, Gibco); MC3T3 cells in α-MEM (Gibco); and U2OS cells in McCoy’s 5A medium (Gibco), each supplemented with 10% fetal bovine serum (Sigma), 1% penicillin–streptomycin (Gibco), and 1% GlutaMAX (Gibco). All cells were maintained in a humidified incubator at 37 °C with 5% CO_2_ and passaged using 0.25% trypsin–EDTA (Gibco) at a split ratio of 1:4 to 1:16. All cell lines were routinely tested for *mycoplasma* contamination by the *mycoplasma* testing team at the Princess Máxima Center.

### STF assay to measure Wnt activity

Wnt/β-catenin signaling activity of the Wnt-IL-13 variants was measured in cell lines (A549, Huh7, MC3T3, and U2OS) containing a stably transfected luciferase reporter gene under the control of a Wnt-responsive promoter (STF reporter). Cells were seeded in 100 μl of culture medium at a density of 1 × 10^5^ cells per well in flat-bottom 96-well plates and incubated overnight at 37 °C with 5% CO_2_. The following day, Wnt-IL-13 variants and the Wnt surrogate without IL-13 fusion (positive control) were added at concentrations ranging from 1.0 nM to 0.164 pM (5-fold serial dilutions) to stimulate Wnt signaling overnight at 37 °C with 5% CO_2_. Anti-FZD antibodies and an anti-LRP5/6 VHH-Fc were included as negative controls. After 20 h of stimulation, the medium was removed, and cells were lysed in 40 μl of 1 × lysis buffer. From each well, 20 μl of lysate was transferred to a white flat-bottom 96-well plate, followed by the addition of 20 μl of luciferase substrate (Promega). Luminescence was measured using a SpectraMax iD3 plate reader (Molecular Devices). The fold induction was calculated according to the formula: Fold induction = (Luminescence_treated_/Luminescence_untreated_). Assays were performed in technical and biological triplicates, and results are presented as the mean ± SD of the normalized values of the biological triplicates in relation to the activity of the Wnt surrogate without IL-13. Dose–response curves were generated in GraphPad Prism 10 (https://www.graphpad.com), and nonlinear regression analysis was applied for curve fitting.

### pSTAT6 assay to measure IL-13 activity

IL-13 signaling activity of the Wnt-IL-13 fusion proteins was assessed in a dose–response assay on HeLa cells by measuring pSTAT6. HeLa cells (1.25 × 10^5^) were seeded in 500 μl of high-glucose complete DMEM (Gibco) in flat-bottom 24-well plates and incubated overnight at 37 °C in 5% CO_2_. The next day, Wnt-IL-13 fusion proteins at different concentrations (0.001 nM to 100 nM, 5-fold serial dilutions) were added to the cells to stimulate IL-13 signaling for 20 min. Subsequently, the cells were washed with PBS, dissociated, collected, and permeabilized with ice-cold methanol (Boom, 100%, v/v) at 4 °C for 15 min. After removing the methanol, the cells were washed, resuspended in fluorescence-activated cell sorting (FACS) buffer, and transferred to V-bottom 96-well plates for staining. Anti-human pSTAT6-PE (CST) and anti-human STAT6-Alexa Fluor 488 (R&D Systems) antibodies were used at a 1:50 dilution, and cells were stained for 45 min at 4 °C. Cells were analyzed by FACS using a Beckman Coulter CytoFLEX S (Beckman Coulter). The extent of pSTAT6 was determined by subtracting the percentage of pSTAT6 cells in the unstimulated samples from that of the stimulated samples. Assays were performed in technical and biological triplicates, and the results are presented as the mean ± SD of the normalized values of the biological triplicates in relation to the activity of recombinant IL-13. Dose–response curves were generated in GraphPad Prism 10, and nonlinear regression analysis was applied for curve fitting.

### Tuft cell differentiation in human ileum organoid

Human ileum AVIL-Clover tuft cell reporter and AVIL lineage-tracing organoids were established and cultured as described in (29). Briefly, organoids were split once a week by mechanical dissociation and cultured in expansion medium as previously described (29). To differentiate tuft cells, human ileum organoids were expanded for 4 days in expansion medium, then organoids were washed for 30 min in DMEM+++: advanced DMEM/F12 (Gibco) supplemented with 100 U ml^−1^ penicillin–streptomycin (Gibco), 10 mM Hepes (Gibco), 1 × GlutaMAX (Gibco). The medium was replaced with tuft cell differentiation medium: 0.5 nM NGS Wnt surrogate (IPA) or 0.5 nM mAb1/mAb2-based Wnt surrogates, 20% (v/v) R-spondin1 (conditioned medium), 10 μM Notch inhibitor DAPT (Sigma-Aldrich), 1 × B-27 Supplement (Life Technologies), 1.25 mM *N*-acetylcysteine (Sigma-Aldrich), and 1% (v/v) recombinant Noggin (IPA). In some experiments, 0.5 nM human IL-13 (Promega) or 0.5 nM Wnt-IL-13 fusion protein was used in this study.

For irradiation of AVIL lineage-tracing organoids (Fig. 3, *F*–*I*), organoids were first differentiated for 4 days in tuft cell differentiation medium containing Wnt-IL-13 fusion protein. Then, they were treated with 1 μM tamoxifen for 20 h, followed by splitting and irradiation (9 Gy) 1 day postsplitting.

### Irradiation in organoids

Culture plates were sealed airtight and irradiated with a single dose of 6 Gy (Fig. 3, *C*–*E*) or 9 Gy (Fig. 3, *F*–*I*) using a linear accelerator (Elekta Precise Linear Accelerator 11F49, Elekta). Plates were placed on a 2-cm thick polystyrene base and submerged in a 37 °C water bath. Irradiation was delivered from below, with the plates positioned exactly 100 cm from the radiation source. Medium was refreshed after irradiation.

### Flow cytometry

Organoids were dissociated into single cells using TrypLE (TrypLE Express, Life Technologies) with 10 μM Rho kinase inhibitor (AbMole) at 37 °C and mechanical disruption by pipetting every 5 min. Then, cells were stained with 4′,6-diamidino-2-phenylindole and visualized using a BD LSR Fortessa X20 4 laser (BD Biosciences, FACSDiva v.9.0, https://www.bdbiosciences.com/en-nl/products/software/instrument-software/bd-facsdiva-software) based on fluorescence levels. FlowJo software (v.10.6.2, https://flowjo.com/previous-versions-flowjo) was used to analyze the flow cytometry data.

### Whole-mount staining of organoids

Organoids were removed from the basement membrane extract (BME) and then were fixed for 30 min at room temperature in formalin. Next, the organoids were permeabilized using 0.1% Tween 20 (Sigma-Aldrich) in PBS for at least 15 min and blocked for at least 1 h in 0.1% Triton X-100 (Sigma-Aldrich), 1 g l^−1^ bovine serum albumin (Sigma-Aldrich) in PBS. Organoids were incubated with Alexa Fluor 488 phalloidin (1:1,000, Thermo Fisher Scientific, A12379) in blocking buffer containing 4′,6-diamidino-2-phenylindole (1:1,000, Invitrogen, D1306). Sections were embedded in fructose–glycerol clearing solution and then visualized on a Leica SP8 confocal microscope (LAS X v.1.1). Image analysis was performed using ImageJ (Fiji, v.1.51n, https://imagej.net/ij/) software.

## Data availability

No new sequencing data were generated as part of this study. To support the main finding of this paper, we reanalyzed single-cell RNA-seq data from the following sources: Ileum-derived human organoids under Wnt and IL-13 signaling (GSE233451, Fig. 1, *A* and *B*), and the Gut Cell Atlas (https://www.gutcellatlas.org/), 10.5061/dryad.8pk0p2ns8, and used for Figure 1, *C* and *D*. Source data are provided with this paper.

## Declaration of Generative AI and AI-Assisted Technologies in the Writing Process

In the final stages of preparing this manuscript, the authors used Grammarly to improve language and grammar. The authors reviewed and edited the content as needed and take full responsibility for the final manuscript.

## Supporting information

This article contains supporting information (8, 22).

## Conflict of interest

H. C. holds several patents related to organoid technology. His full disclosure can be found at https://www.uu.nl/staff/JCClevers/AncillaryActivities. C. Y. J. holds patents on the Wnt surrogate technology and is a minor shareholder of Surrozen, a company developing Wnt surrogate-based therapeutics; she was a cofounder of the company but no longer has an active role. The other authors declare that they have no conflicts of interest with the contents of this article.
