# Supplementary material for: A dual-pathway Wnt-IL-13 fusion protein enhances human intestinal regeneration through tuft cell activation
Source: J Biol Chem. 2026 May 24;302(7):113187. doi: 10.1016/j.jbc.2026.113187 (PMC13311165; doi:10.1016/j.jbc.2026.113187)
Supplement: Appendix [file mmc1.docx]

# Appendix A

***Table: Overview of the constructs used for transfections and their characteristics****. In the amino acid sequences the FZD-binding antibodies (mAb1 and mAb2) with light chain (LC) and heavy chain (HC) highlighted in blue, the anti-LRP5/6 VHH in green and the human IL13 in orange. Several constructs form the base of other constructs, restriction enzymes used for this purpose are mentioned in the right column.*

| **Description and code** | **Amino acid sequence** |
| --- | --- |
| mAb2 LC  (343) | DIQMTQSPSSLSASVGDRVTITCRASQSVSSAVAWYQQKPGKAPKLLIYSASSLYSGVPSRFSGSRSGTDFTLTISSLQPEDFATYYCQQGVYLFTFGQGTKVEIKRTVAAPSVFIFPPSDEQLKSGTASVVCLLNNFYPREAKVQWKVDNALQSGNSQESVTEQDSKDSTYSLSSTLTLSKADYEKHKVYACEVTHQGLSSPVTKSFNRGEC |
| mAb2 LC with N-terminal VHH  (344) | EVQLQESGGGLVQAGGSLRLSCAASGRTFSIYTIGWFRQAPGKEREFVAEITWSGGSTYYADSVKGRFTISRDNAKNTVYLQMNSLKPEDTAVYYCAAITYTRGIYKYWGQGTQVTVSSGSGSGGSGSGDIQMTQSPSSLSASVGDRVTITCRASQSVSSAVAWYQQKPGKAPKLLIYSASSLYSGVPSRFSGSRSGTDFTLTISSLQPEDFATYYCQQGVYLFTFGQGTKVEIKRTVAAPSVFIFPPSDEQLKSGTASVVCLLNNFYPREAKVQWKVDNALQSGNSQESVTEQDSKDSTYSLSSTLTLSKADYEKHKVYACEVTHQGLSSPVTKSFNRGEC |
| mAb2 HC  (352) | EVQLVESGGGLVQPGGSLRLSCAASGFNIHSSSIHWVRQAPGKGLEWVAATYSSFGSITYADSVKGRFTISADTSKNTAYLQMNSLRAEDTAVYYCARYHHPFGYALDYWGQGTLVTVSSASTKGPSVFPLAPSSKSTSGGTAALGCLVKDYFPEPVTVSWNSGALTSGVHTFPAVLQSSGLYSLSSVVTVPSSSLGTQTYICNVNHKPSNTKVDKKVEPKSCDKTHTCPPCPAPEAAGGPSVFLFPPKPKDTLMISRTPEVTCVVVDVSHEDPEVKFNWYVDGVEVHNAKTKPREEQYNSTYRVVSVLTVLHQDWLNGKEYKCKVSNKALPAPIEKTISKAKGQPREPQVYTLPPSREEMTKNQVSLTCLVKGFYPSDIAVEWESNGQPENNYKTTPPVLDSDGSFFLYSKLTVDKSRWQQGNVFSCSVMHEALHNHYTQKSLSLSPGK |
| mAb2 LC with N-terminal IL-13 and VHH  (387) | PGPVPPSTALRELIEELVNITQNQKAPLCNGSMVWSINLTAGMYCAALESLINVSGCSAIEKTQRMLSGFCPHKVSAGQFSSLHVRDTKIEVAQFVKDLLLHLKKLFREGRFNGSGGSGGGGSEVQLQESGGGLVQAGGSLRLSCAASGRTFSIYTIGWFRQAPGKEREFVAEITWSGGSTYYADSVKGRFTISRDNAKNTVYLQMNSLKPEDTAVYYCAAITYTRGIYKYWGQGTQVTVSSGSGSGGSGSGDIQMTQSPSSLSASVGDRVTITCRASQSVSSAVAWYQQKPGKAPKLLIYSASSLYSGVPSRFSGSRSGTDFTLTISSLQPEDFATYYCQQGVYLFTFGQGTKVEIKRTVAAPSVFIFPPSDEQLKSGTASVVCLLNNFYPREAKVQWKVDNALQSGNSQESVTEQDSKDSTYSLSSTLTLSKADYEKHKVYACEVTHQGLSSPVTKSFNRGEC |
| mAb2 LC with N-terminal VHH and C-terminal IL-13  (388) | EVQLQESGGGLVQAGGSLRLSCAASGRTFSIYTIGWFRQAPGKEREFVAEITWSGGSTYYADSVKGRFTISRDNAKNTVYLQMNSLKPEDTAVYYCAAITYTRGIYKYWGQGTQVTVSSGSGSGGSGSGDIQMTQSPSSLSASVGDRVTITCRASQSVSSAVAWYQQKPGKAPKLLIYSASSLYSGVPSRFSGSRSGTDFTLTISSLQPEDFATYYCQQGVYLFTFGQGTKVEIKRTVAAPSVFIFPPSDEQLKSGTASVVCLLNNFYPREAKVQWKVDNALQSGNSQESVTEQDSKDSTYSLSSTLTLSKADYEKHKVYACEVTHQGLSSPVTKSFNRGECGSGGSGGGGSPGPVPPSTALRELIEELVNITQNQKAPLCNGSMVWSINLTAGMYCAALESLINVSGCSAIEKTQRMLSGFCPHKVSAGQFSSLHVRDTKIEVAQFVKDLLLHLKKLFREGRFN |
| mAb 2 HC with N-terminal IL-13  (389) | PGPVPPSTALRELIEELVNITQNQKAPLCNGSMVWSINLTAGMYCAALESLINVSGCSAIEKTQRMLSGFCPHKVSAGQFSSLHVRDTKIEVAQFVKDLLLHLKKLFREGRFNGSGGSGGGGSEVQLVESGGGLVQPGGSLRLSCAASGFNIHSSSIHWVRQAPGKGLEWVAATYSSFGSITYADSVKGRFTISADTSKNTAYLQMNSLRAEDTAVYYCARYHHPFGYALDYWGQGTLVTVSSASTKGPSVFPLAPSSKSTSGGTAALGCLVKDYFPEPVTVSWNSGALTSGVHTFPAVLQSSGLYSLSSVVTVPSSSLGTQTYICNVNHKPSNTKVDKKVEPKSCDKTHTCPPCPAPEAAGGPSVFLFPPKPKDTLMISRTPEVTCVVVDVSHEDPEVKFNWYVDGVEVHNAKTKPREEQYNSTYRVVSVLTVLHQDWLNGKEYKCKVSNKALPAPIEKTISKAKGQPREPQVYTLPPSREEMTKNQVSLTCLVKGFYPSDIAVEWESNGQPENNYKTTPPVLDSDGSFFLYSKLTVDKSRWQQGNVFSCSVMHEALHNHYTQKSLSLSPGK |
| mAb2 HC with C-terminal IL-13  (390) | EVQLVESGGGLVQPGGSLRLSCAASGFNIHSSSIHWVRQAPGKGLEWVAATYSSFGSITYADSVKGRFTISADTSKNTAYLQMNSLRAEDTAVYYCARYHHPFGYALDYWGQGTLVTVSSASTKGPSVFPLAPSSKSTSGGTAALGCLVKDYFPEPVTVSWNSGALTSGVHTFPAVLQSSGLYSLSSVVTVPSSSLGTQTYICNVNHKPSNTKVDKKVEPKSCDKTHTCPPCPAPEAAGGPSVFLFPPKPKDTLMISRTPEVTCVVVDVSHEDPEVKFNWYVDGVEVHNAKTKPREEQYNSTYRVVSVLTVLHQDWLNGKEYKCKVSNKALPAPIEKTISKAKGQPREPQVYTLPPSREEMTKNQVSLTCLVKGFYPSDIAVEWESNGQPENNYKTTPPVLDSDGSFFLYSKLTVDKSRWQQGNVFSCSVMHEALHNHYTQKSLSLSPGKGSGGSGGGGSPGPVPPSTALRELIEELVNITQNQKAPLCNGSMVWSINLTAGMYCAALESLINVSGCSAIEKTQRMLSGFCPHKVSAGQFSSLHVRDTKIEVAQFVKDLLLHLKKLFREGRFN |
| mAb1 HC  (392) | EVQLVQSGAEVKKPGASVKVSCKASGYTFTSYGISWVRQAPGQGLEWMGWISAYNGNTNYAQKLQGRVTMTTDTSTSTAYMELRSLRSDDTAVYYCASSKEKATYYYGMDVWGQGTTVTVSSASTKGPSVFPLAPSSKSTSGGTAALGCLVKDYFPEPVTVSWNSGALTSGVHTFPAVLQSSGLYSLSSVVTVPSSSLGTQTYICNVNHKPSNTKVDKKVEPKSCDKTHTCPPCPAPEAAGGPSVFLFPPKPKDTLMISRTPEVTCVVVDVSHEDPEVKFNWYVDGVEVHNAKTKPREEQYNSTYRVVSVLTVLHQDWLNGKEYKCKVSNKALGAPIEKTISKAKGQPREPQVYTLPPSREEMTKNQVSLTCLVKGFYPSDIAVEWESNGQPENNYKTTPPVLDSDGSFFLYSKLTVDKSRWQQGNVFSCSVMHEALHNHYTQKSLSLSPGK |
| mAb1 LC with N-terminal VHH  (394) | EVQLQESGGGLVQAGGSLRLSCAASGRTFSIYTIGWFRQAPGKEREFVAEITWSGGSTYYADSVKGRFTISRDNAKNTVYLQMNSLKPEDTAVYYCAAITYTRGIYKYWGQGTQVTVSSGGGGSGSGSGQAVVLQEPSLSVSPGGTVTLTCGLSSGSVSTNYYPSWYQQTPGQAPRTLIYYTNTRSSDVPERFSGSIVGNKAALTITGAQPDDESVYFCLLYLGRGIWVFGGGTKLTVLGQPKAAPSVTLFPPSSEELQANKATLVCLISDFYPGAVTVAWKADSSPVKAGVETTTPSKQSNNKYAASSYLSLTPEQWKSHRSYSCQVTHEGSTVEKTVAPTECS |
| mAb1 LC with N-terminal IL-13 and VHH  (395) | PGPVPPSTALRELIEELVNITQNQKAPLCNGSMVWSINLTAGMYCAALESLINVSGCSAIEKTQRMLSGFCPHKVSAGQFSSLHVRDTKIEVAQFVKDLLLHLKKLFREGRFNGSGGSGGGGSEVQLQESGGGLVQAGGSLRLSCAASGRTFSIYTIGWFRQAPGKEREFVAEITWSGGSTYYADSVKGRFTISRDNAKNTVYLQMNSLKPEDTAVYYCAAITYTRGIYKYWGQGTQVTVSSGSGSGQAVVLQEPSLSVSPGGTVTLTCGLSSGSVSTNYYPSWYQQTPGQAPRTLIYYTNTRSSDVPERFSGSIVGNKAALTITGAQPDDESVYFCLLYLGRGIWVFGGGTKLTVLGQPKAAPSVTLFPPSSEELQANKATLVCLISDFYPGAVTVAWKADSSPVKAGVETTTPSKQSNNKYAASSYLSLTPEQWKSHRSYSCQVTHEGSTVEKTVAPTECS |
| mAb1 LC with N-terminal VHH and C-terminal IL-13  (396) | EVQLQESGGGLVQAGGSLRLSCAASGRTFSIYTIGWFRQAPGKEREFVAEITWSGGSTYYADSVKGRFTISRDNAKNTVYLQMNSLKPEDTAVYYCAAITYTRGIYKYWGQGTQVTVSSGGGGSGSGSGQAVVLQEPSLSVSPGGTVTLTCGLSSGSVSTNYYPSWYQQTPGQAPRTLIYYTNTRSSDVPERFSGSIVGNKAALTITGAQPDDESVYFCLLYLGRGIWVFGGGTKLTVLGQPKAAPSVTLFPPSSEELQANKATLVCLISDFYPGAVTVAWKADSSPVKAGVETTTPSKQSNNKYAASSYLSLTPEQWKSHRSYSCQVTHEGSTVEKTVAPTECSGSGGSGGGGSPGPVPPSTALRELIEELVNITQNQKAPLCNGSMVWSINLTAGMYCAALESLINVSGCSAIEKTQRMLSGFCPHKVSAGQFSSLHVRDTKIEVAQFVKDLLLHLKKLFREGRFN |
| mAb1 HC with N-terminal IL-13  (397) | PGPVPPSTALRELIEELVNITQNQKAPLCNGSMVWSINLTAGMYCAALESLINVSGCSAIEKTQRMLSGFCPHKVSAGQFSSLHVRDTKIEVAQFVKDLLLHLKKLFREGRFNGSGGSGGGGSEVQLVQSGAEVKKPGASVKVSCKASGYTFTSYGISWVRQAPGQGLEWMGWISAYNGNTNYAQKLQGRVTMTTDTSTSTAYMELRSLRSDDTAVYYCASSKEKATYYYGMDVWGQGTTVTVSSASTKGPSVFPLAPSSKSTSGGTAALGCLVKDYFPEPVTVSWNSGALTSGVHTFPAVLQSSGLYSLSSVVTVPSSSLGTQTYICNVNHKPSNTKVDKKVEPKSCDKTHTCPPCPAPEAAGGPSVFLFPPKPKDTLMISRTPEVTCVVVDVSHEDPEVKFNWYVDGVEVHNAKTKPREEQYNSTYRVVSVLTVLHQDWLNGKEYKCKVSNKALGAPIEKTISKAKGQPREPQVYTLPPSREEMTKNQVSLTCLVKGFYPSDIAVEWESNGQPENNYKTTPPVLDSDGSFFLYSKLTVDKSRWQQGNVFSCSVMHEALHNHYTQKSLSLSPGK |
| mAb1 HC with C-terminal IL-13  (398) | EVQLVQSGAEVKKPGASVKVSCKASGYTFTSYGISWVRQAPGQGLEWMGWISAYNGNTNYAQKLQGRVTMTTDTSTSTAYMELRSLRSDDTAVYYCASSKEKATYYYGMDVWGQGTTVTVSSASTKGPSVFPLAPSSKSTSGGTAALGCLVKDYFPEPVTVSWNSGALTSGVHTFPAVLQSSGLYSLSSVVTVPSSSLGTQTYICNVNHKPSNTKVDKKVEPKSCDKTHTCPPCPAPEAAGGPSVFLFPPKPKDTLMISRTPEVTCVVVDVSHEDPEVKFNWYVDGVEVHNAKTKPREEQYNSTYRVVSVLTVLHQDWLNGKEYKCKVSNKALGAPIEKTISKAKGQPREPQVYTLPPSREEMTKNQVSLTCLVKGFYPSDIAVEWESNGQPENNYKTTPPVLDSDGSFFLYSKLTVDKSRWQQGNVFSCSVMHEALHNHYTQKSLSLSPGKGSGGSGGGGSPGPVPPSTALRELIEELVNITQNQKAPLCNGSMVWSINLTAGMYCAALESLINVSGCSAIEKTQRMLSGFCPHKVSAGQFSSLHVRDTKIEVAQFVKDLLLHLKKLFREGRFN |
| mAb1 LC  (700) | QAVVLQEPSLSVSPGGTVTLTCGLSSGSVSTNYYPSWYQQTPGQAPRTLIYYTNTRSSDVPERFSGSIVGNKAALTITGAQPDDESVYFCLLYLGRGIWVFGGGTKLTVLQPKAAPSVTLFPPSSEELQANKATLVCLISDFYPGAVTVAWKADSSPVKAGVETTTPSKQSNNKYAASSYLSLTPEQWKSHRSYSCQVTHEGSTVEKTVAPTECS |
| Fc-VHH  (716) | EPKSSDKTHTCPPCPAPELLGGPSVFLFPPKPKDTLMISRTPEVTCVVVDVSHEDPEVKFNWYVDGVEVHNAKTKPREEQYNSTYRVVSVLTVLHQDWLNGKEYKCKVSNKALPAPIEKTISKAKGQPREPQVYTLPPSREEMTKNQVSLTCLVKGFYPSDIAVEWESNGQPENNYKTTPPVLDSDGSFFLYSKLTVDKSRWQQGNVFSCSVMHEALHNHYTQKSLSLSPGAGSGSGHHHHHHGSGLEVLFQGPGEVQLQESGGGLVQAGGSLRLSCAASGRTFSIYTIGWFRQAPGKEREFVAEITWSGGSTYYADSVKGRFTISRDNAKNTVYLQMNSLKPEDTAVYYCAAITYTRGIYKYWGQGTQVTVSS |
