# Supplementary material for: A dual-pathway Wnt-IL-13 fusion protein enhances human intestinal regeneration through tuft cell activation
Source: J Biol Chem. 2026 May 24;302(7):113187. doi: 10.1016/j.jbc.2026.113187 (PMC13311165; doi:10.1016/j.jbc.2026.113187)
Supplement: Supporting Figures [file mmc3.pdf]

Suppl. Figure 1

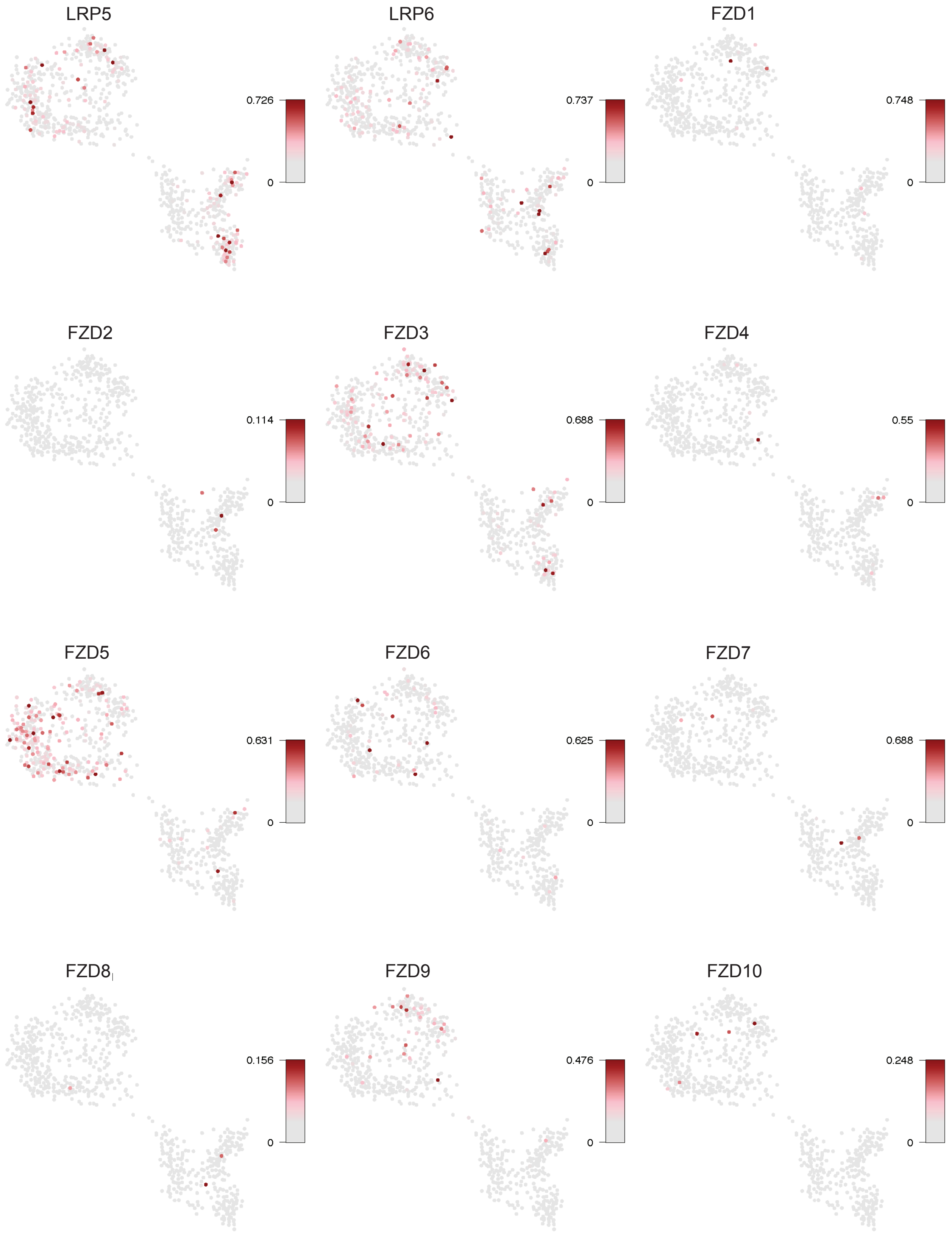

Supplementary Figure 1: FZD and LRP5/6 expression in ileum-derived organoids

Log-normalized expression of the ten FZDs and LRP5/6, projected on a Metacell 2D representation of scRNA-seq data of ileum-derived organoids cultured in tuft cell differentiation media with or without IL-4/IL-13 activation, as in Figure 1A[8].

Suppl. Figure 2

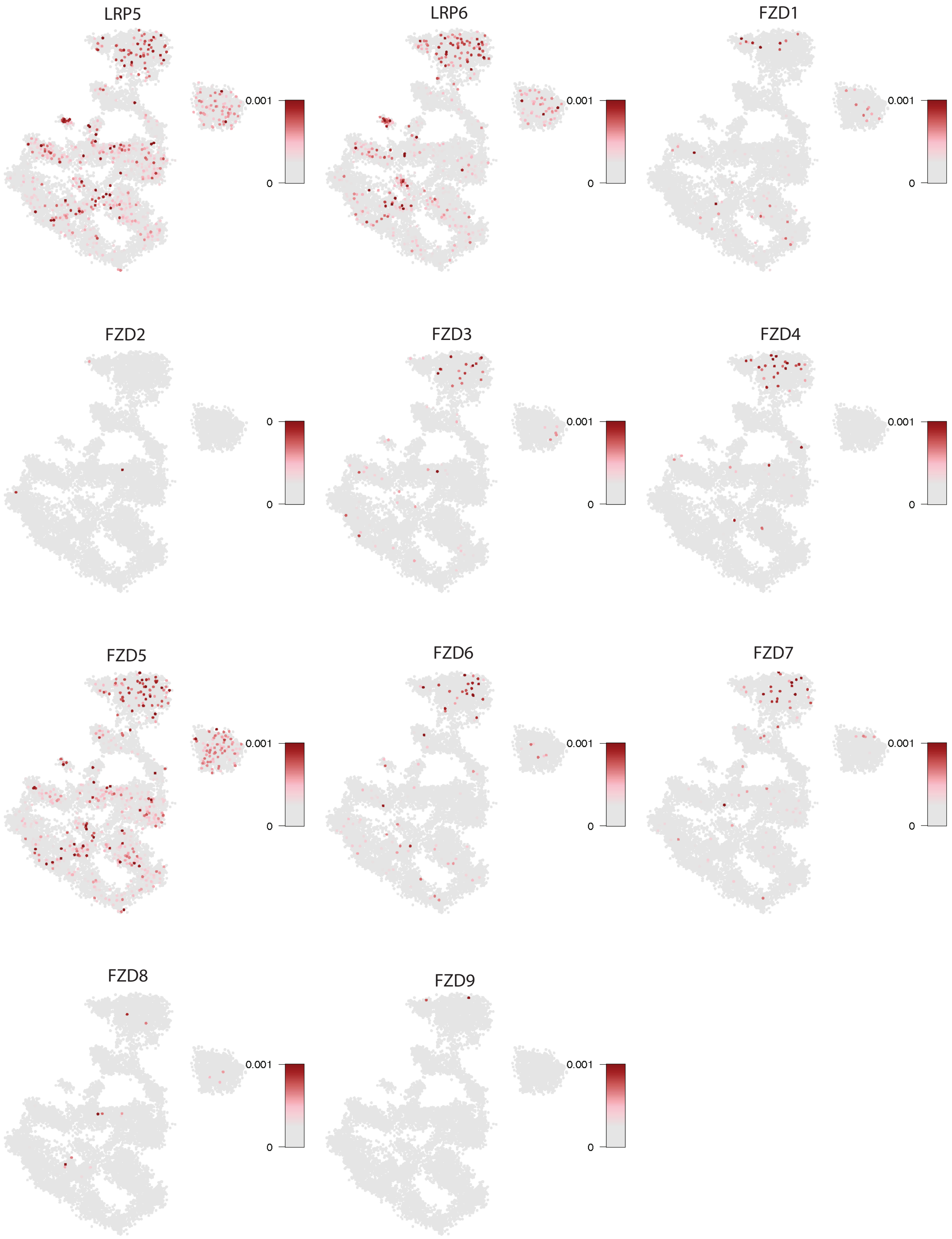

Supplementary Figure 2: FZD and LRP5/6 expression in human adult small intestine

Log-normalized expression of the ten FZDs and LRP5/6, projected on a Metacell 2D representation of scRNA-seq data of primary human adult small intestine epithelial cells, as in Figure 1C[22].

Suppl. Figure 3

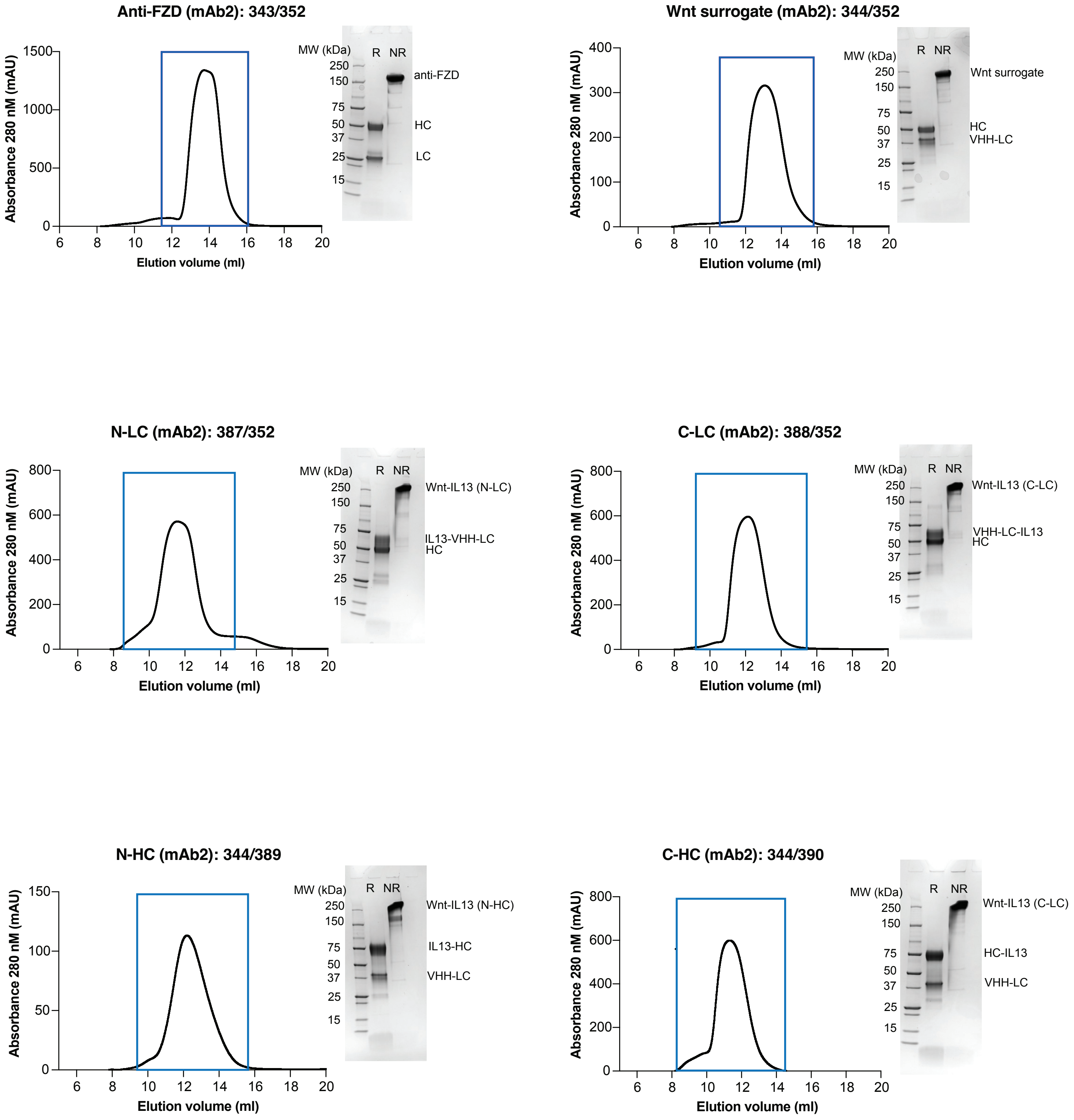

Supplementary Figure 3: Characterization of the mAb2-based Wnt-IL-13 fusion proteins

Size-exclusion chromatography (SEC) traces of mAb2-based Wnt-IL-13 fusion proteins used in functional assays. The pooled fractions are indicated (blue box). Numbers indicated above the SEC traces correspond to the amino acid sequences for the heavy and light chains as provided in the Appendix. SDS-PAGE analysis (4-20%) of the pooled samples under reducing and non-reducing conditions confirms the integrity of the fusion proteins and the expected heavy and light chain composition.

Suppl. Figure 4

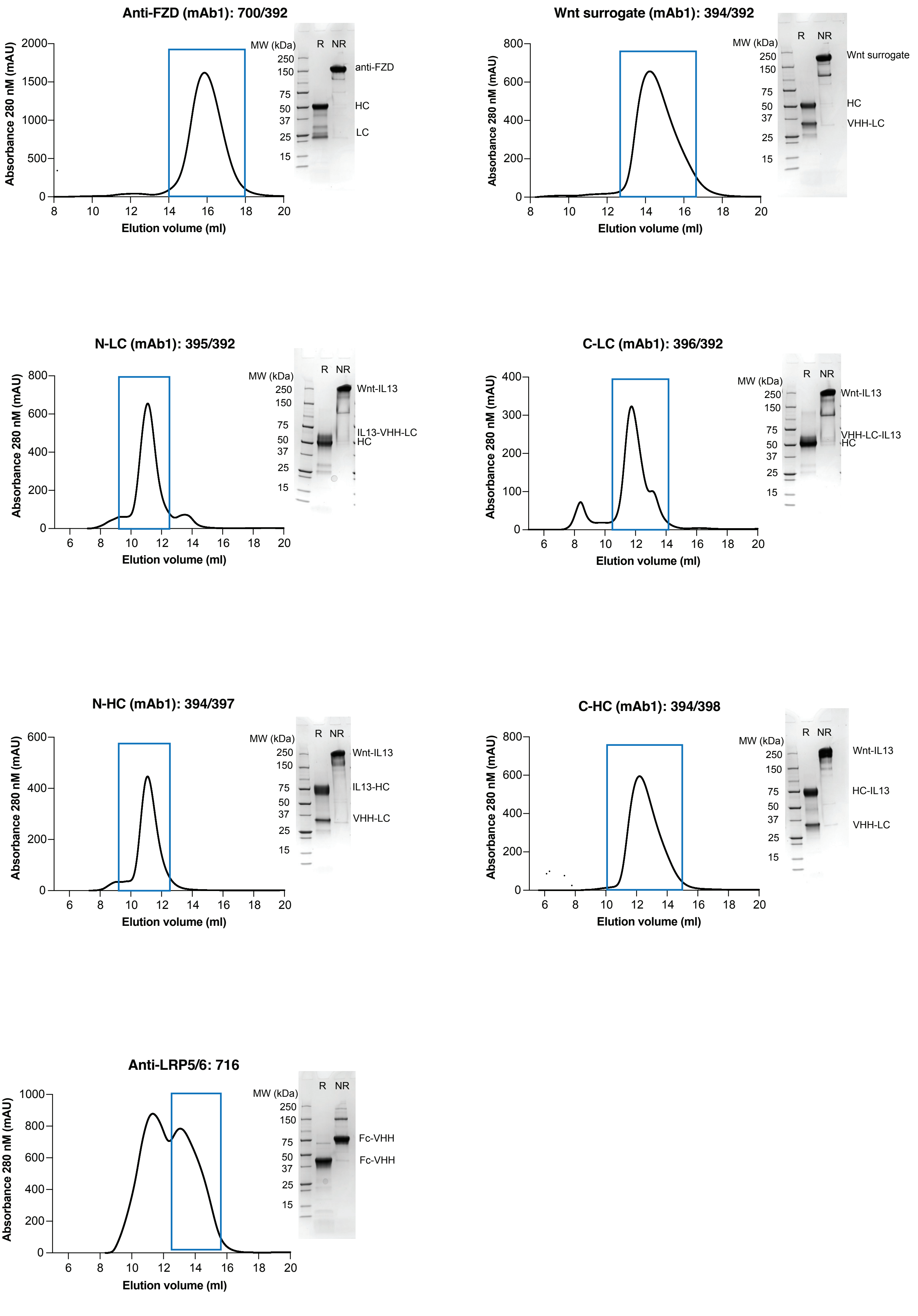

Supplementary Figure 4: Characterization of the mAb1-based Wnt-IL-13 fusion proteins

Size-exclusion chromatography (SEC) traces of mAb1-based Wnt-IL-13 fusion proteins used in functional assays. The pooled fractions are indicated (blue box). Numbers indicated above the SEC traces correspond to the amino acid sequences for the heavy and light chains as provided in the Appendix. SDS-PAGE analysis (4-20%) of the pooled samples under reducing and non-reducing conditions confirms the integrity of the fusion proteins and the expected heavy and light chain composition.

Suppl. Figure 5

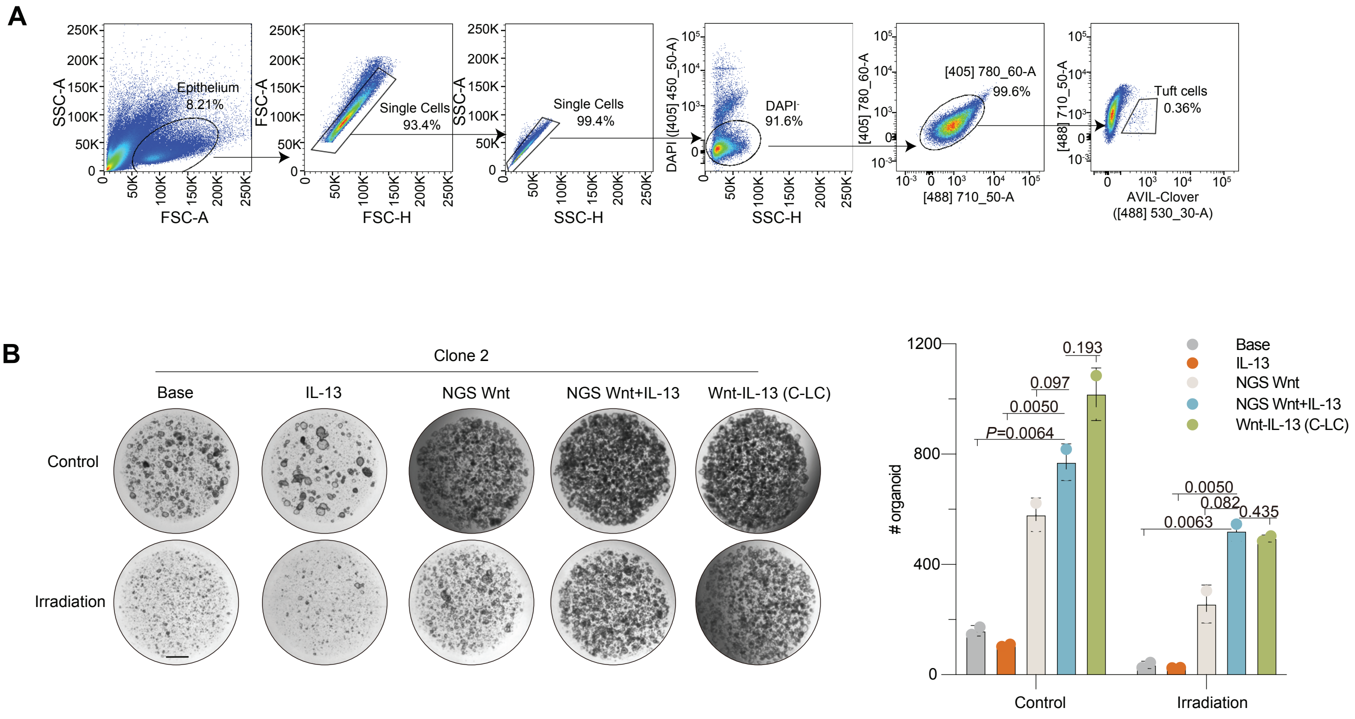

Supplementary Figure 5: Wnt-IL-13 fusion proteins in human intestine organoids

(A) Gating strategy for flow cytometric analysis of AVIL-Clover reporter organoids.

(B) Representative images (left) and quantification (right) of organoid numbers from the second organoid clone cultured in different regimes following irradiation (related to Figure 3C). **Left:** Scale bar, 1 mm. **Right**: Each dot is one BME drop. n=2 drops per condition. Data are presented as mean ± SD. *P* values are derived from false discovery rate (FDR)-adjusted unpaired two-tailed Student's *t*-test against the NGS Wnt +IL-13 group.
